# Supplementary material for: Neural encoding of perceived patch value during competitive and hazardous virtual foraging
Source: Nat Commun. 2021 Sep 16;12:5478. doi: 10.1038/s41467-021-25816-9 (PMC8446065; doi:10.1038/s41467-021-25816-9)
Supplement: Supplementary file 1 — Supplementary Information [file 41467_2021_25816_MOESM1_ESM.pdf]

Neural encoding of perceived patch value during competitive and hazardous virtual foraging

Supplementary Information

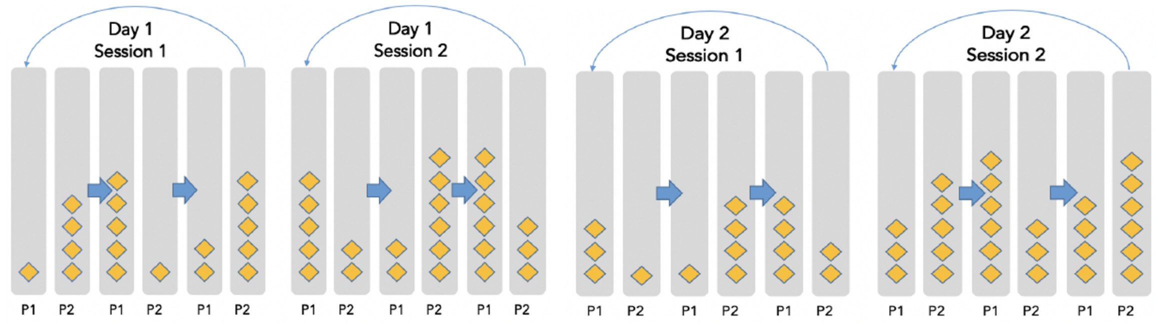

Supplementary Figure 1

Task design competitor states and cycles. The design featured two sessions each day over two days, in which patch configurations changed in repeating cycles; each diamond represents a competitor such that, for example, on Day 1 Session the first patch state included one competitor in P1 and four competitors in patch 2; P1 (patch 1); P2 (patch 2), followed by a state in which P1 contained five competitors and P2 contained one competitor, followed by a state in which P1 contained two competitors and P2 contained five competitors. P1: Patch 1; P2: Patch 2.

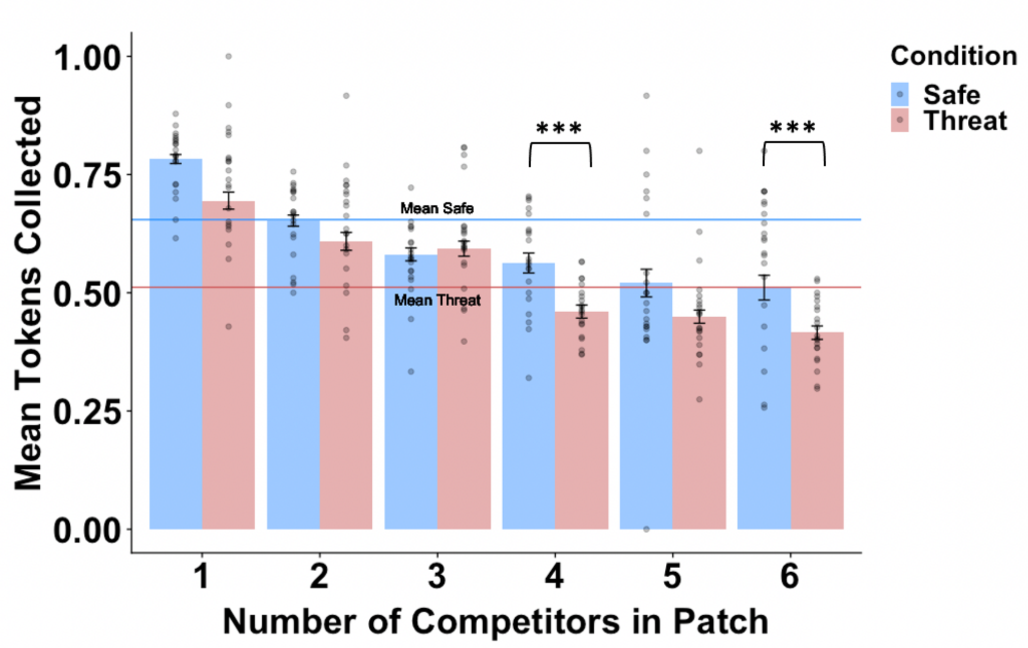

Supplementary Figure 2

Rewards acquired by condition and number of competitors. Mean token collection across competitor number in selected patch across conditions. s The average difference favored the safe condition, especially in patches with higher numbers of competitors. Horizontal lines display overall averages for token collection for each condition. Patches with four (4) (two-sample t-test; p=0.0004), and six (6) (two-sample t-test; p=0.0004) competitors demonstrated significant differences in reward collection between conditions. Errors bars represent standard error of the mean. A sample of n=20 was used to derive error bar statistics.

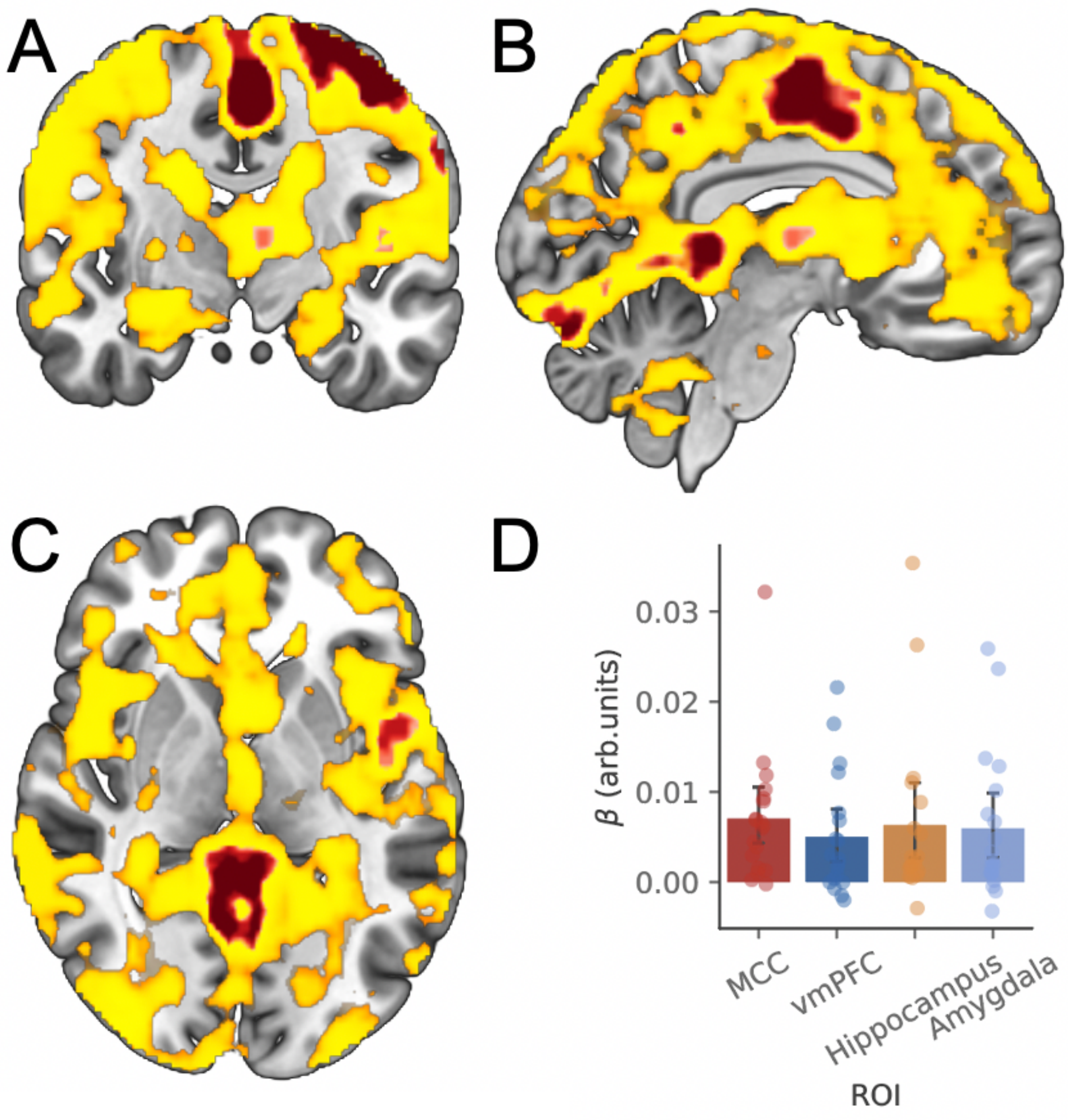

Supplementary Figure 3

RSA results. Panels A, B, C: Regions representing threat versus safety in the RSA analysis. The map represents p values determined using threshold-free cluster correction (TFCE) , two-sided. Yellow/orange shows areas where $p < .05$, while red shows areas where $p < .001$, with color intensity reflecting the strength of the effect. Panel D: Values from the mid-cingulate cortex (MCC), ventromedial prefrontal cortex (vmPFC), hippocampus and amygdala. These are provided for illustration only; statistical tests were performed on whole-brain data. Error bars represent 95% confidence intervals across 19 participants, and the center represents the mean. arb. units = arbitrary units; ROI = region of interest.

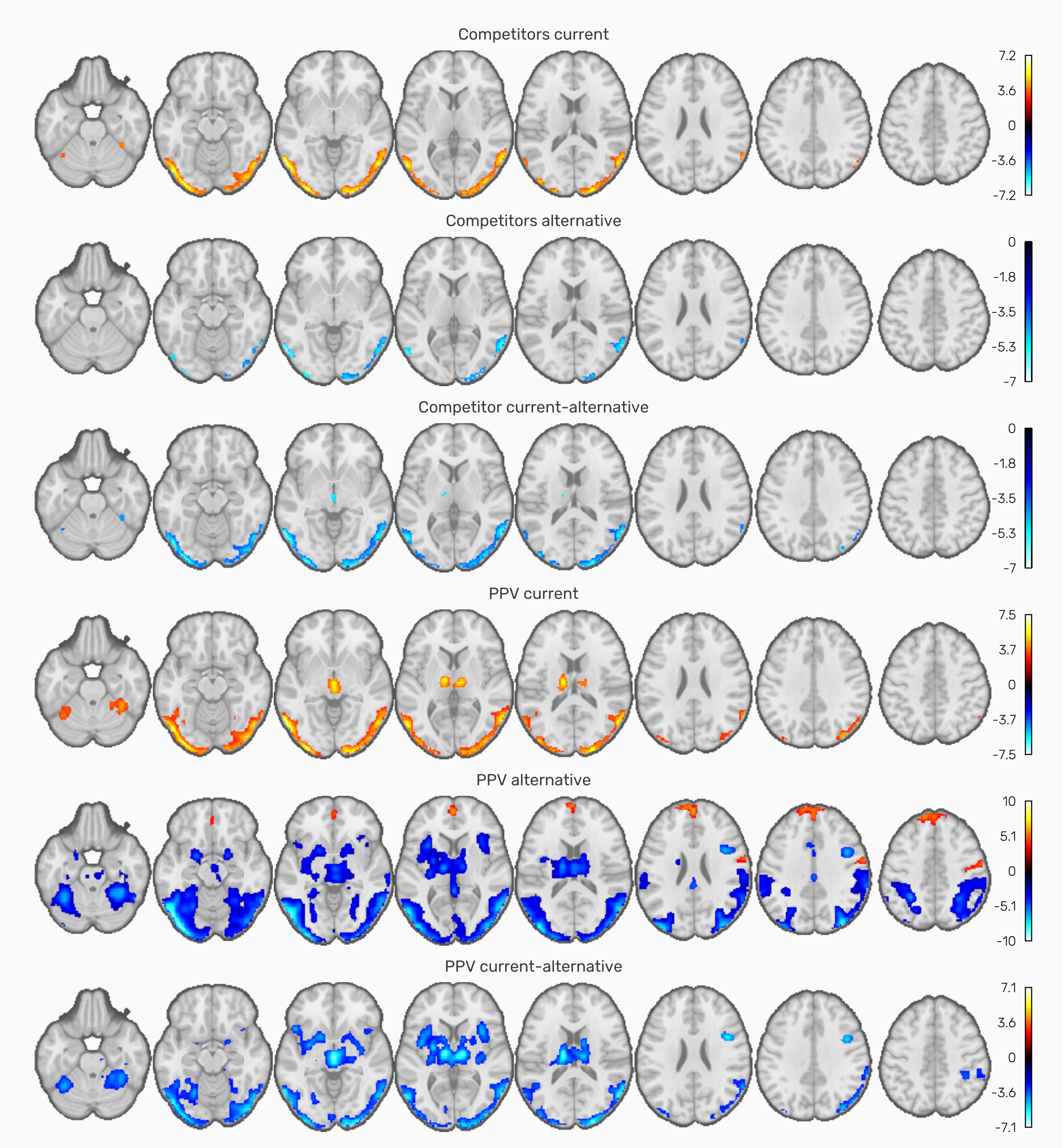

Supplementary Figure 4

Univariate analysis results for PPV, current and alternative patches. Results of univariate analyses, showing effects for variables of interest. Values represent t-statistics and maps are thresholded at $p < .05$ TFCE corrected, two-sided. PPV: Perceived patch value.

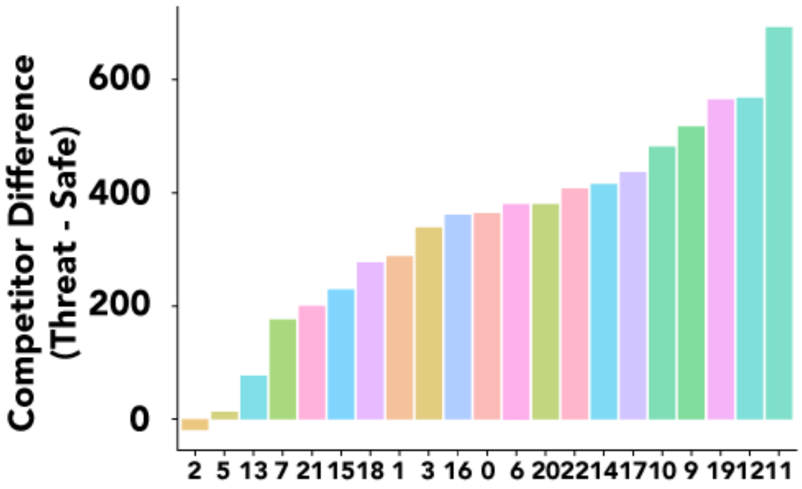

Supplementary Figure 5

Individual variability in competition difference. Each bar represents an individual participant, by participant number. Bar level indicates total competitors for selected patches in the threat condition minus total competitors for selected patches in the safe condition. High bars indicate extreme threat avoidance, while bars closer to 0 indicate no sensitivity to threat.

	Comp. diff	Current comp.	Alternative comp.	Threat	SV diff	Current SV	Alternative SV
Comp. diff							
Current comp.	0.65						
Alternative comp.	0.65	0.25					
Threat	-0.01	-0.02	0.00				
SV diff	0.21	0.18	0.20	0.21			
Current SV	0.09	0.19	0.00	0.67	0.49		
Alternative SV	0.11	-0.01	0.22	0.64	0.51	0.43	

Supplementary Table 1

RDM correlations used in RSA analysis. Pearson correlations between representational dissimilarity matrices (RDMs) used in the RSA analysis. Values represent the mean correlation across participants.

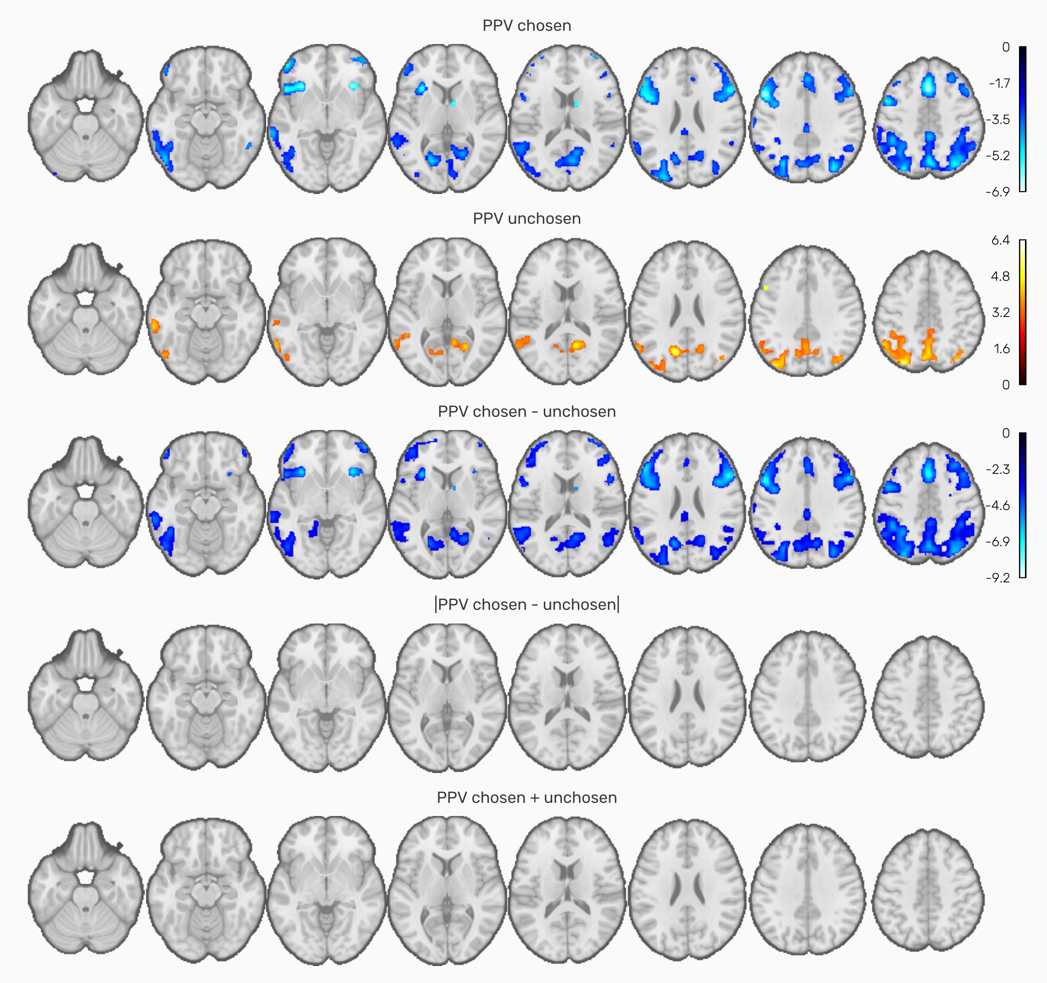

Supplementary Figure 6

Univariate results comparing chosen and unchosen patches. Results of univariate analyses focused on value of the chosen and unchosen patches. Values represent t-statistics and maps are thresholded at $p < .05$ TFCE corrected, two-sided. PPV: Perceived patch value.
