# Supplementary material for: Neural encoding of perceived patch value during competitive and hazardous virtual foraging
Source: Nat Commun. 2021 Sep 16;12:5478. doi: 10.1038/s41467-021-25816-9 (PMC8446065; doi:10.1038/s41467-021-25816-9)
Supplement: Supplementary file 3 — Reporting Summary [file 41467_2021_25816_MOESM3_ESM.pdf]

nature research

Corresponding author(s): Dean Mobbs

Last updated by author(s): Jun 9, 2021

Reporting Summary

Nature Research wishes to improve the reproducibility of the work that we publish. This form provides structure for consistency and transparency in reporting. For further information on Nature Research policies, see our Editorial Policies and the Editorial Policy Checklist.

Statistics

For all statistical analyses, confirm that the following items are present in the figure legend, table legend, main text, or Methods section.

n/a	Confirmed	
☐	☒	The exact sample size (n) for each experimental group/condition, given as a discrete number and unit of measurement
☐	☒	A statement on whether measurements were taken from distinct samples or whether the same sample was measured repeatedly
☐	☒	The statistical test(s) used AND whether they are one- or two-sided<br>*Only common tests should be described solely by name; describe more complex techniques in the Methods section.*
☐	☒	A description of all covariates tested
☐	☒	A description of any assumptions or corrections, such as tests of normality and adjustment for multiple comparisons
☐	☒	A full description of the statistical parameters including central tendency (e.g. means) or other basic estimates (e.g. regression coefficient) AND variation (e.g. standard deviation) or associated estimates of uncertainty (e.g. confidence intervals)
☒	☐	For null hypothesis testing, the test statistic (e.g. F, t, r) with confidence intervals, effect sizes, degrees of freedom and P value noted<br>*Give P values as exact values whenever suitable.*
☐	☒	For Bayesian analysis, information on the choice of priors and Markov chain Monte Carlo settings
☐	☒	For hierarchical and complex designs, identification of the appropriate level for tests and full reporting of outcomes
☒	☐	Estimates of effect sizes (e.g. Cohen's d, Pearson's r), indicating how they were calculated

Our web collection on statistics for biologists contains articles on many of the points above.

Software and code

Policy information about availability of computer code

Data collection	Python 2.7x was used to write the foraging game program, including the aesthetic design, task parameters and data collection mechanism.
Data analysis	fMRI data was preprocessed using fMRI Prep version stable RRID:SCR_016216, including aCompCorm; motion corrected using mcflirt (FSL v5.0.9; brain tissue segmentation of CSF, white matter and gray matter was performed using fast (FSL v5.0.9). T1w volume was corrected for INU (intensity non-uniformity) using N4BiasFieldCorrection34 v2.1.0 and skull-stripped using antsBrainExtraction.sh v2.1.0 (using the OASIS template). Spatial normalization to the ICBM 152 Nonlinear Asymmetrical template version 2009c35 [RRID:SCR_008796] was performed through nonlinear registration with the antsRegistration tool of ANTs v2.1.036 [RRID:SCR_004757]. Motion correcting transformations, BOLD-to-T1w transformation and T1w-to-template (MNI) warp were concatenated and applied in a single step using antsApplyTransforms (ANTs v2.1.0). First level and neural RSA models were constructed using FSL 6.0. Behavioral analysis was performed using the R language v3.5.0, and Python 3.7. Study code can be accessed here: https://github.com/mobbslab/foraging_paper; and via the DOI: 10.5281/zenodo.5113171

For manuscripts utilizing custom algorithms or software that are central to the research but not yet described in published literature, software must be made available to editors and reviewers. We strongly encourage code deposition in a community repository (e.g. GitHub). See the Nature Research guidelines for submitting code & software for further information.

Data

Policy information about availability of data

All manuscripts must include a data availability statement. This statement should provide the following information, where applicable:

- Accession codes, unique identifiers, or web links for publicly available datasets
- A list of figures that have associated raw data
- A description of any restrictions on data availability

The behavioral and neural datasets generated during and/or analyzed herein are openly available and stored in online repositories. Behavioral data and analysis

code are available at https://github.com/mobbslab/foraging_paper and DOI: 10.5281/zenodo.5113171. The code provided on github supported all panels associated with Figure 2. Neuroimaging data used to perform the neural modeling can be accessed at https://openneuro.org/datasets/ds003484.

Field-specific reporting

Please select the one below that is the best fit for your research. If you are not sure, read the appropriate sections before making your selection.

☐ Life sciences ☒ Behavioural & social sciences ☐ Ecological, evolutionary & environmental sciences

For a reference copy of the document with all sections, see nature.com/documents/nr-reporting-summary-flat.pdf

Behavioural & social sciences study design

All studies must disclose on these points even when the disclosure is negative.

Study description	This study is a quantitative study utilizing a within subjects design assessing behavioral and neural responses to threatening and non-threatening aspects of a virtual environment. Participants completed the same task, in the same order. Participants were asked to select among two patch options, and then forage within the selected patch for food items. Some trials included a threat, represented by a virtual predator, that could arrive at any time and end the trial. If the player was captured they received a light electric shock and lost a portion of their resources.
Research sample	Sample was a convenience sample, derived via SONA systems in Pasadena CA for healthy participants aged 18-49, 6F, 18M, average age 31. Sample is believed to be representative.
Sampling strategy	Convenience sample near the CalTech campus. Number of participants was determined based on task parameters and data set size for each participant. fMRI scanning time approached 3 hours per participant; behavioral data points approached 600 per participant.
Data collection	Data was automatically collected using an fMRI scanner, the Python game program, and Qualtrics for surveys. No person was present inside the scanning room. The experimenter and technician (when applicable) observed from a distinct room. The experimenter administered the task, which drew on 576 files each representing an individual trial. The experimenters were aware of the conditions and hypotheses, however all instructions were provided on screen with no interaction required by the experimenter during the task.
Timing	10/12/19 - 3/15/20
Data exclusions	Exclusions occurred for two (2) participants for which either behavioral or neural, or both types of data were lost due to computer malfunctions.
Non-participation	No drop outs
Randomization	All participants received the same task consisting of all conditions.

Reporting for specific materials, systems and methods

We require information from authors about some types of materials, experimental systems and methods used in many studies. Here, indicate whether each material, system or method listed is relevant to your study. If you are not sure if a list item applies to your research, read the appropriate section before selecting a response.

Materials & experimental systems

n/a	Involved in the study	
☒	☐	Antibodies
☒	☐	Eukaryotic cell lines
☒	☐	Palaeontology and archaeology
☒	☐	Animals and other organisms
☐	☒	Human research participants
☒	☐	Clinical data
☒	☐	Dual use research of concern

Methods

n/a	Involved in the study	
☒	☐	ChIP-seq
☒	☐	Flow cytometry
☐	☒	MRI-based neuroimaging

Human research participants

Policy information about studies involving human research participants

Population characteristics	See above.
Recruitment	Recruitment was conducted using the SONA recruitment system around the California Institute of Technology campus. The sample included both student and non-students. SONA participant pools typically consist of individuals that are actively seeing work, and hence compensation, or those interested in particular areas of science.
Ethics oversight	Statement added to Participants section of manuscript: All participants provided informed consent to participate in the study, which was reviewed and approved by the Committee for the Protection of Human Subjects (IRB) at the California Institute of Technology institutional review board.

Note that full information on the approval of the study protocol must also be provided in the manuscript.

Magnetic resonance imaging

Experimental design

Design type	Task; Event-Related
Design specifications	4 blocks of 144 trials each; ~5 sec between trials; each block took about 50 minutes to complete
Behavioral performance measures	Decisions, tokens collected, shocks received, x/y coordinates. Sums, means, s.e. were calculated for each variable of interest to assess compliance with task demands.

Acquisition

Imaging type(s)	functional imaging (fMRI)
Field strength	3T
Sequence & imaging parameters	single-shot, multiband T2*-weighted echo planar imaging sequence with the following parameters: TR/TE = 1000/30 ms, Flip Angle = 60°, 72 slices, slice angulation = 20° to transverse, multiband acceleration = 6, no in-plane acceleration, 3/4 partial Fourier acquisition, slice thickness/gap = 2.0/0.0 mm, FOV = 192 mm × 192 mm, matrix = 96 × 96). Anatomical reference imaging will employ 0.9 mm isotropic resolution 3D T1w MEMP-RAGE (TR/TI/TE = 2550/1150/1.3, 3.1, 4.0, 6.9 ms, FOV = 230 m x 230 mm) and 3D T2w SPACE sequences (TR/TE = 3200/564 ms, FOV = 230 mm x 230 mm).
Area of acquisition	Whole Brain

Diffusion MRI ☐ Used ☒ Not used

Preprocessing

Preprocessing software	Preprocessing was conducted using fMRIPrep, a standardized preprocessing pipeline for fMRI.
Normalization	Data were normalized to the MNI152 template.
Normalization template	The MNI152 template was used for normalization.
Noise and artifact removal	6 motion parameters (3 translation, 3 rotation) in addition to signal extracted from white matter and CSF were included as covariates in the first-level analysis.
Volume censoring	No volume censoring was employed

Statistical modeling & inference

Model type and settings	A fixed effects mass-univariate approach was used at the first level to derive beta maps for each trial. This was then used for representational similarity analysis based on the similarity of these maps across trials.
Effect(s) tested	RSA was used to investigate representations of socially-adjusted value and the number of competitors alone, in addition to the difference in these between patches, in addition to threat level (safe versus risk of shock).

Specify type of analysis: ☒ Whole brain ☐ ROI-based ☐ Both

Statistic type for inference (See Eklund et al. 2016)	Clusterwise using threshold-free cluster correction and 5000 permutations, thresholded at $p < .05$
Correction	See above.

Models & analysis

n/a	Involved in the study
☒	☐ Functional and/or effective connectivity
☒	☐ Graph analysis
☐	☒ Multivariate modeling or predictive analysis

Multivariate modeling and predictive analysis	We used models conducted in PyMC3 to estimate socially-adjusted value based on estimates obtained via Bayesian models that took into account rewards collected, shock risk and the individual level cost value for receiving a shock. We also used a softmax function that transformed the values of the two patch options into choice probabilities in order to predict decisions (left or right patch).
